# All-Solid-State Lithium Battery Working without an Additional Separator in a Polymeric Electrolyte

**DOI:** 10.3390/polym10121364

**Published:** 2018-12-09

**Authors:** Seonggyu Cho, Shinho Kim, Wonho Kim, Seok Kim, Sungsook Ahn

**Affiliations:** 1 Secondary Battery R&D Center, DRB Holdings Co., Pusan 46329, Korea; cho.seong.gyu@drbworld.com (S.C.); kim.shin.ho@drbworld.com (S.K.); 2 Department of Chemical and Biochemical Engineering, Pusan National University, Pusan 46241, Korea; whkim@pnu.edu (W.K.); seokkim@pusan.ac.kr (S.K.); 3 Department of Mechanical Engineering, Pohang University of Science and Technology (POSTECH), Pohang 37673, Korea

**Keywords:** secondary Li ion battery, all-solid-state battery, solid polymer electrolyte, succinonitrile (SN), lithium(trifluoromethanesulfonyl)imide (LiTFSI)

## Abstract

Considering the safety issues of Li ion batteries, an all-solid-state polymer electrolyte has been one of the promising solutions. Achieving a Li ion conductivity of a solid-state electrolyte comparable to that of a liquid electrolyte (>1 mS/cm) is particularly challenging. Even with characteristic ion conductivity, employment of a polyethylene oxide (PEO) solid electrolyte has not been sufficient due to high crystallinity. In this study, hybrid solid electrolyte (HSE) systems have been designed with Li_1.3_Al_0.3_Ti_0.7_(PO_4_)_3_ (LATP), PEO and lithium bis(trifluoromethanesulfonyl)imide (LiTFSI). A hybrid solid cathode (HSC) is also designed using LATP, PEO and lithium cobalt oxide (LiCoO_2_, LCO)—lithium manganese oxide (LiMn_2_O_4_, LMO). The designed HSE system has 2.0 × 10^−4^ S/cm (23 °C) and 1.6 × 10^−3^ S/cm (55 °C) with a 6.0 V electrochemical stability without an additional separator membrane introduction. In these systems, succinonitrile (SN) has been incorporated as a plasticizer to reduce crystallinity of PEO for practical all-solid Li battery system development. The designed HSC/HSE/Li metal cell in this study operates without any leakage and short-circuits even under the broken cell condition. The designed HSC/HSE/Li metal cell in this study displays an initial charge capacity of 82/62 mAh/g (23 °C) and 123.4/102.7 mAh/g (55 °C). The developed system overcomes typical disadvantages of internal resistance induced by Ti ion reduction. This study contributes to a new technology development of all-solid-state Li battery for commercial product design.

## 1. Introduction

Li ion batteries have been credited for a great revolution of the strong intermittent renewable-energy sources replacing fossil fuels. They also critically contribute to the development of communication and transportation by the rise of super-slim smart phones and electric cars in a practical range. However, since announced in 1991, heels of Li ion battery phones and laptops have been recalled because of a flame causing injury to the users [1]. The cycle life is limited because of poor cycling efficiency of Li electrode. The secondary cells are more sensitive to impurities such as water in the electrolyte and the electrode materials. In addition, the cells under running would have Li dendrites leading to occasional explosions. Nonetheless, because of radical needs for high energy density and reliability of batteries, Li technology has been ceaselessly focused on. With high energy density and large capacity suitable for energy storage systems, Li ion is almost unique among all other negative electrode materials that have ever been investigated.

The typical Li ion batteries consist of a negative anode, a positive cathode and a liquid electrolyte. However, many energy storage devices based on combustible organic solvents inevitably carry the risks of leakage, heavier packaging and related hazards. A liquid electrolyte is volatile at high temperature when the battery is charged or discharged quickly or packs of car batteries are damaged in accidents. Against inherent disadvantages of liquid electrolytes [2,3], solid electrolytes of nonflammable polymers [4,5] and all-solid-state batteries using inorganic materials [6,7] have been developed. When a battery releases power, Li ions move from the anode through the electrolyte to the cathode. In this point, more conductive electrolyte generates better battery performance. Therefore, the promise of Li ion solid-state batteries is the replacement of the heavy and sometimes dangerous liquid electrolyte with lighter, more versatile and solid alternatives. Although finding a solid electrolyte with conductivity comparable to liquids has been still a challenge, the solid electrolyte batteries are popular. They are safer because flammable components are removed and deliver more power by replacing the carbon-based anodes with Li metal. This leads to a higher energy density and cycle life with less weight and cost. In addition, without a need to struggle to safely pack a liquid electrolyte in a shape, all-solid-state batteries can be fabricated in more versatile shapes, reducing manufacturing costs. This could make electric cars a more enticing proposition with longer running distance and a lower purchase price. In many ways, all-solid-state constructions certainly enhance the overall performance of energy storage and conversion [8,9,10,11,12,13,14].

A potential solution against the disadvantage of solid-state electrolytes has been pursued in this point [15,16,17,18,19]. The conductivity of polymer electrolytes such as polyethylene oxide (PEO) has been utilized [20,21,22,23], which is considered by the electron donating activity, coordinate bonding and hopping of Li ions [24]. As other electrolytes, alkali crystalline complexes of PEO with metals have also been investigated with prominent ion conductivity [25,26,27,28]. However, the Li agent in PEO is not sufficient to generate effective ionic conductivity because high crystallinity of PEO impairs Li ion movement. The amorphous sites of PEO contribute to enhance the Li ion movement especially at room temperature [29,30,31,32]. When the PEO complex with relatively low glass transition temperature (*T*_g_) becomes amorphous, the ion conductivity is 10^−5^–10^−4^ S/cm, corresponding to 3–4 times higher than those of crystallite counterparts. However, the PEO complex displays a liquid-like property, which simultaneously induces short-circuits, the typical disadvantages of liquids.

### 1.1. Oxide Solid Electrolytes

Inorganic oxides have been designed for electrolyte components [33,34,35,36], such as Perovskite-structured Li_3x_La_2/3−x_TiO_3_ [33], Garnet-structured Li_7_La_3_Zr_2_O_12_ (LLZO) [34,35], NASICON-structured Li_1.3_Al_0.3_Ti_0.7_(PO_4_)_3_(LATP) [36]. Nonetheless, low conductivity, high grain boundary resistance and high calcination temperature of these oxide solid electrolytes cause increased volatility, phase transition and impurity, decreasing the total quality of Li ion battery. On the other hand, sulfide-based solid electrolyte including Li_10_GeP_2_S_12_ (LGPS) display high conductivity almost similar to that of liquid electrolytes [37]. However, it is smelly, expensive, unstable under air and reactive with water, generating harmful H_2_S. A battery system composed of lithium aluminum germanium phosphate (LAGP, Li_1.5_Al_0.5_Ge_1.5_(PO_4_)_3_), and PEO has been tried with protecting layers [38]. In addition, it works at around 50 °C but not at lower room temperature. One of the oxide solid electrolytes, lithium aluminum titanium phosphate (LATP), is highly stable under air and is inexpensive. However, LATP exhibits lower ion conductivity than liquid electrolytes or sulfide-based electrolytes, reduction of Ti^4+^ into Ti^3+^ by the interaction of Li ion, and high grain boundary resistance. In addition, the lattice structure of solid electrolytes modified by Ti ion reduction affects the Li ion movement. The complex composed of Ti^4+^ typically generates a conductivity close to 10^−3^ S/cm while that of Ti^3+^ significantly decreases the conductivity to 10^−7^ S/cm [39]. To overcome these problems, protecting thin layers composed of PEO or Al_2_O_3_ has been introduced to minimize the interaction with Li ion [38,40]. An LATP pellet was dip-coated by PEO for a protecting layer formation between the LATP and Li metal surface where the whole system can exhibit ionic conductivity of 5.03 × 10^−6^ S/cm at 23 °C [41]. In this study, new battery systems are designed without protecting layer introduction, which also effectively work at room temperature.

### 1.2. Succinonitrile (SN) Introduction

Lithium bis(trifluoromethanesulfonyl)imide (LiTFSI) exhibits limitation in narrow electrochemical windows (~4.8 V) and low electrochemical stability. To overcome these disadvantages, nonionic polymeric crystalline with excellent electrochemical stability is obtained by using succinonitrile (SN) [42,43]. Nitrile compounds are typically safe against fire and chemically stable under harsh conditions. SN has a *T*_g_ of −40 °C and a melting temperature (*T*_m_) of 55 °C. Between these temperatures, SN suppresses the crystallization of PEO and increases the physical stability against short-circuit. In addition, SN is highly polar to enhance the Li dissolution and movement. When SN forms a complex with LiTFSI, the conductivity increases up to 1 × 10^−3^ S/cm (25 °C) due to molecular rotation and trans-gauche isomerism [44,45]. Even though LGPS is relatively expensive and reactive with water, generating unfavorable H_2_S [46], an LGPS+PEO+SN hybrid system does not affect Li ion movement due to SN contribution [43]. The chemical reactivity of Li ions with LATP determines the ionic conductivity, but this has not been fully investigated for LATP+PEO+SN hybrid systems, and there is a lack of systematic electrochemical analysis to verify the interaction with Li ions.

In this study, all-solid-state Li ion batteries using designed hybrid solid electrolytes (HSEs) are investigated to overcome the typical disadvantages of PEO-based Li ion battery systems. Compromising the properties of LATP by overcoming the disadvantageous grain boundary resistance by PEO introduction, SN is utilized for increased mechanical property and reduced possibility of short-circuits. Ion conductivity is prominently enhanced at room temperature by using effective dissolution of an SN complex with LiTFSI and PEO. The chemical reaction is monitored by the day-by-day changes in impedance considering the physical interaction of LATP and Li metal. The reduction of Ti ion is investigated by using X-ray photoelectron spectroscopy (XPS) after the charging-discharging repetition. Linear scanning voltammetry (LSV) curves of the designed electrodes confirm the extended region of electrochemical windows of PEO-only system which is originally narrow, near 4.8 V.

## 2. Experimental Section

### 2.1. Chemicals

All chemicals were purchased and used without further purification: Conductive carbon black (TIMCAL Graphite & Carbon Super P^®^ Conductive Carbon Black, MTI Corporation, Richmond, CA, USA), Li_2_CO_3_ (lithium carbonate, 99%, Sigma-Aldrich, St. Louis, MO, USA), Al_2_O_3_ (aluminum oxide, 99.99%, Sigma-Aldrich, St. Louis, MO, USA),TiO_2_ (Cotiox KA-100, 98%, Cosmo Chemical, Incheon, Korea), NH_4_H_2_PO_4_ (ammonium dihydrogenphosphate, 99.999%, Sigma-Aldrich, St. Louis, MO, USA), PEO (polyethylene oxide, *M*_W_ = 1 × 10^5^, 3 × 10^5^ and 6 × 10^5^, Sigma-Aldrich, St. Louis, MO, USA), LiTFSI (bis(trifluoromethane) sulfonimide lithium salt, 99.95%, Sigma-Aldrich, St. Louis, MO, USA), LiPF_6_ (lithium hexafluorophosphate, 99.99%, Sigma-Aldrich, St.Louis, MO, USA), AN (acetonitrile anhydrous, 99.8%, Sigma-Aldrich, St. Louis, MO, USA), SN (succinonitrile, 99%, Sigma-Aldrich, St. Louis, MO, USA), LCO (PoscoESM, Gumi, Korea), LMO (G05, ILJIN materials, Seoul, Korea).

### 2.2. Synthesis of LATP

The solid electrolyte LATP (Li_1.3_Al_0.3_Ti_0.7_(PO_4_)_3_) is synthesized by a conventional solid-state method. Stoichiometric amounts of Li_2_CO_3_, Al_2_O_3_, TiO_2_, and (NH_4_)H_2_PO_4_ are mixed in a ball mill for 12 h. The slurry is sintered in a furnace at 900 °C for 2 h. The dried powder is then jet milled at 1800 rpm for 2 h to reduce the particle size. D-values (D_10_, D_50_ & D_90_) are measured for particle size evaluation as an intercept for 10%, 50% and 90% of the cumulative mass and D_50_~ 6 μm is obtained for effective mixing process.

### 2.3. Preparation of Hybrid Solid Electrolytes (HSE) and Hybrid Solid Electrolyte Cathode (HSC)

The Li ion conducting polymer PEO is mixed with LiTFSI ([EO]:[Li] = 8:1) and previously synthesized LATP (LATP:PEO = 8:2 by weight) in acetonitrile (AN). The polymer solution is mixed using a centrifugal mixer (THINKY mixer ARM-310, Oxfordshire, UK) at 2000 rpm for 15 min, with or without 5 wt % of SN (Succinonitrile). The slurry is mixed again for 15 min. For the tests, this HSE slurry is casted on Al foil for analysis or on an electrode for coin cell test. After natural drying at room temperature for 1 h, samples are dried completely in a 50 °C vacuum oven for 12 h.

**Figure 1:**
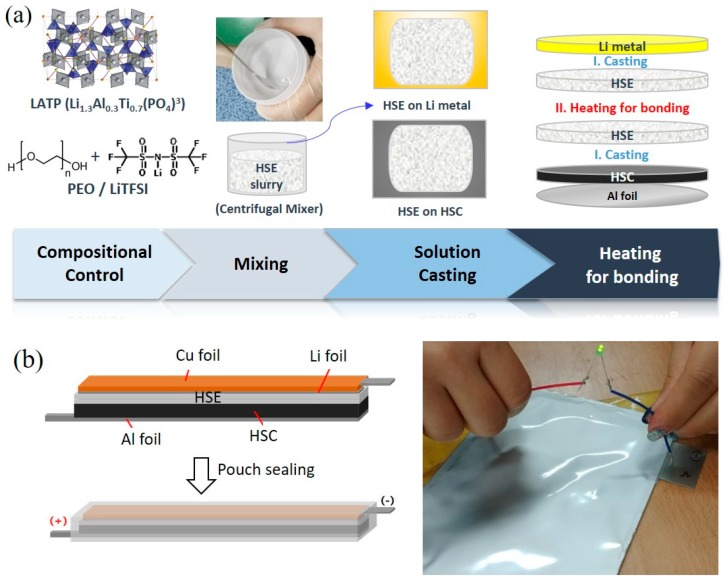
(**a**) Preparation of all-solid-state coin-cell using the designed HSE and HSC in this study. (**b**) Configuration of all-solid-state pouch cell at a 5 × 10 cm^2^ scale (**left** scheme) and its operation (**right** picture).

For better binding in the electrode, higher molecular weight PEO (M_w_ ~3 × 10^5^ and 6 × 10^5^) was employed. LATP, PEO (LATP:PEO = 5:5 by weight) and LiTFSI ([EO]:[Li] = 10:1) were mixed in AN using THINKY mixer for 15 min. LiMn_2_O_4_ and LiCoO_2_ as active materials and Super-P as conductive material were added in additional solvent AN, followed by mixing for 15 min. After the HSC slurry was casted on Al foil by Scalpel, the electrodes were dried at 80 °C in a vacuum oven for 24 h. HSE slurry was casted on the dried electrode and Li metal. The aforementioned HSE and HSC thickness is designed uniformly at 270 μm and 80 μm scale and the active material loading of electrodes is designed uniformly at a 10 mg cm^−2^ (±0.1 mg cm^−2^) scale. The HSC/HSE composite was dried under the aforementioned condition. Differing from the cathode side, the Li metal/HSE composite was dried at room temperature for 24 h to prevent crumbling. To evaluate the electrochemical performance, the CR2032 coin-type cell and all-solid-state pouch cell at a 5 × 10 cm^2^ scale was fabricated with HSC/HSE composite and Li metal/HSE by a lamination process. The construction of the cell is described as Figure 1. All processes were conducted in a dry-room.

### 2.4. Characterizations

Synthesized LATP and the HSC/HSE composites were observed using a scanning electron microscope (SEM, SEC Mini SEM SNE-3000M, Pleasanton, CA, USA) and X-ray diffraction (XRD, Rigaku MiniFlex 600, The Woodlands, TX, USA). XRD patterns of samples were obtained over a 2θ range from 10 deg to 80 deg with Cu Kα radiation at room temperature. The scan rate was 6 deg/min. Linear sweep voltammetry (LSV) was performed for both oxidation and reduction procedures for selected samples using a potentiostat (Bio Logic SP-150) with scan rate of 20 mV/s from OCV to 1.5 V or 6.0 V. Ionic conductivity is calculated by the AC impedance method with symmetric SS/Al/HSE/Al/SS cell using a multi-channel potentiostat (Bio Logic VMP3, Seyssinet-Pariset, France). The samples were placed at 25 °C and 55 °C for 12 h, and then analyzed from 500 kHz to 1 Hz at open-circuit voltage with 5.0 mV amplitude. With a differential scanning calorimeter (DSC, DISCOVERY DSC 2500, TA Instrument, New Castle, DE, USA), the calorimetric measurement is performed from room temperature to 100 °C at the heating rate of 10 °C/min. For each C-rate (1C = 1.5 mA cm^−2^), the cell was charged using a constant current charge (0.05C & each C-rate) using a charge step from 3.0 V to 4.3 V, followed by a constant voltage charge at 4.3 V. For discharge, the constant current discharge (0.05C & each C-rate) from 4.3 V to 3.0 V was applied at room temperature or 55 °C.

## 3. Results

### 3.1. Design of Hybrid Solid Electrolytes (HSE) and Hybrid Solid Cathode (HSC)

This study introduces a designed hybrid solid electrolyte (HSE) and hybrid solid cathode (HSC). The HSCs are designed by combining lithium manganese oxide (LMO, LiMn_2_O_4_) with lithium cobalt oxide (LCO, LiCoO_2_). In Table 1, composition and ion conductivity of the designed HSC and HSE are summarized at two temperature conditions (23 and 55 °C). Li ion-conducting PEO is mixed with lithium aluminum titanium phosphates (LATP) synthesized in this study (Experimental Section). This sticky white HSE slurry is casted on Al or Li metal foil. For better casting process, the viscosity of the slurry is modified by controlling the content of LATP and PEO as well as molecular weight of PEO. The battery efficiency is compared with lithium(trifluoromethanesulfonyl)imide (LiTFSI) and lithium hexafluorophosphate (LiPF_6_). In addition, the function of SN is evaluated in terms of the interaction with PEO and LiTFSI. Ion conductivity is compared with pure PEO electrolyte (HSE-1) and PEO/LATP (20/80) composite electrolyte with LiPF_6_ (HSE-2). With LiTFSI, the PEO/LATP (20/80) composite electrolyte without (HSE-3) and with (HSE-4) SN incorporation is compared. Two types of LMO/LCO (30/30)-based cathodes are compared containing the PEO/LATP (12.8/12.8) composite combined with LiTFSI, without (HSC-1) and with (HSC-2) SN incorporation.

A representative coin cell preparation using the designed HSE-4 and HSC-2 is illustrated in Figure 1a. The slurry is coated on Li metal foil by solution casting. LATP, PEO, LiTFSI and SN are mixed in a designed composition using a centrifugal mixer (Thinky Mixer) resulting in a sticky white HSE-4 slurry. LMO and LCO are mixed with PEO and SN, resulting in HSC-2. The HSC-2 is coated on Al foil, followed by HSE-4 slurry casting. The Li metal anode and the designed cathode plates are bonded by heating HSE sides to form a layered structure of a coin cell. In addition, this process is scaled-up for a pouch cell (5 × 10 cm^2^) in which copper foil is additionally employed outside of the Li metal plate for physical protection (Figure 1b). The designed all-solid-state lithium pouch battery is successfully working in spite of its breakage without any leakage and short-circuits (Supporting Information, Movie S1).

### 3.2. Structure of the All-Solid-State Battery System

**Figure 2:**
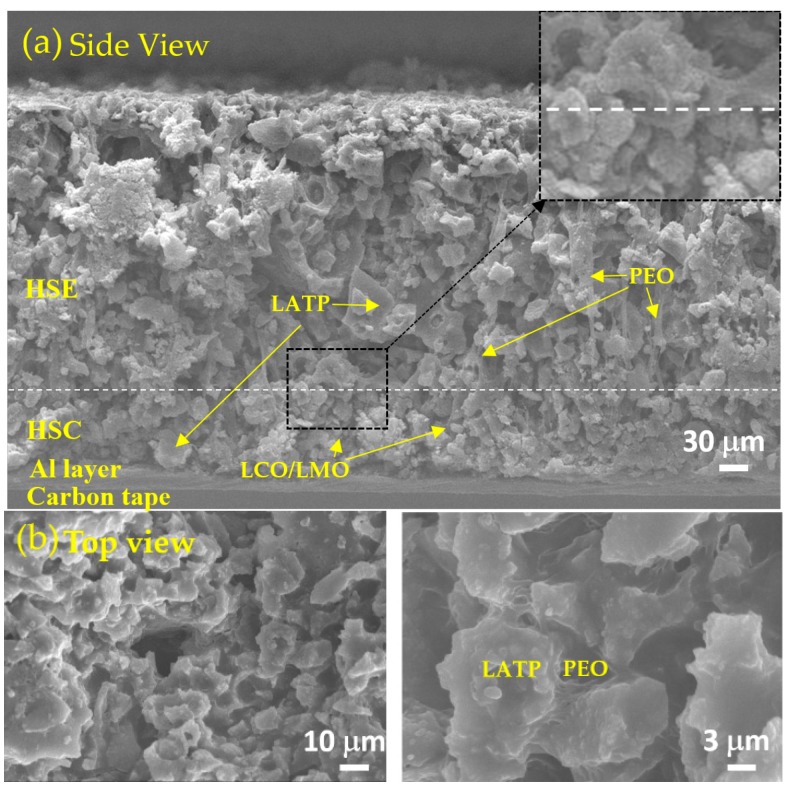

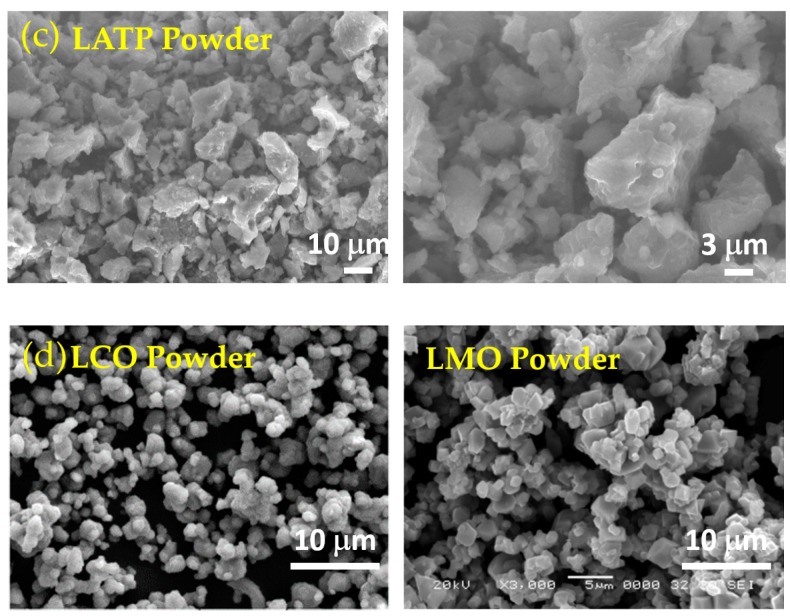
SEM images of (**a**) cross-section and (**b**) Top view of HSE-4/HSC-2/Al foil on carbon tape. SEM images of (**c**) LATP powder and (**d**) LCO and LMO powder. Scale bars in the images.

The simplified battery sample is prepared to investigate the performance of HSE and HSC, which are casted and dried on a layered structure stacked from Al foil, cathode (HSC-2) and electrolyte (HSE-4) layers without anode Li metal and protective Cu foil layers. A representative scanning electron microscopy (SEM) image of each layer is shown in a cross-sectional side-view (Figure 2a) and in a top-view (Figure 2b). In the cross-sectional image, the size of particulates in HSC layer (below the dotted-line in the picture) is smaller compared with that in the HSE layer; this is caused by the relatively small size of the LCO and LMO particles (Figure 2d). The images of pure LATP powder are compared in Figure 2c. The top-view images of the designed battery system (Figure 2b) are similar to those of the LATP powder pellet (Figure 2c). For all the systems, the inter-particulate spaces are effectively filled with PEO binding. The SEM images confirm effective physical contact of ion-conducting PEO and solid LATP in the designed HSE layer without phase separation, which is crucial for effective ionic conductivity of a battery. Figure 2b depicts the reticulated PEO connects LATP particles forming ion-conducting pathways, which can decrease boundary resistance in the solid LATP electrolyte. All of these morphologies are advantageous for the increase in the bulk ionic conductivity [47,48,49].

### 3.3. Conductivity of the Designed Systems

The total resistance is evaluated by ionic conductivity based on the electrochemical impedance spectroscopy (EIS) (Table 1). Using the measured resistance and following relation, ionic conductivity (*σ*, S/cm) of the designed HSE and HSC is evaluated,

(1) $$\sigma =\frac{l}{A\cdot S}$$

where *l* is the thickness (cm) of the HSE, *A* is the area (cm^2^) of the sample, *S* is the total resistance (Ω) obtained from EIS spectra. The ionic conductivity of the designed HSE shows almost three orders higher (~10^−4^ S/cm) than that of pure PEO (10^−6^~10^−8^ S/cm) [50] at the same EO to Li (8:1). This result confirms the contribution of high ionic conductivity of LATP embedded in the ion-conducting PEO by the reduced bulk and boundary resistance. Due to characteristic temperature-dependence, ionic conductivity at 55 °C is about 10 times higher than that at 23 °C in every sample. The composite system consisting of LiTFSI shows similar ionic conductivity as that using LiPF_6_. By adding SN, ionic conductivities (2.0 × 10^−4^ S/cm @ 23 °C & 1.6 × 10^−3^ S/cm @ 55 °C) are enhanced compared with those (1.5 × 10^−4^ S/cm @ 23 °C & 1.4 × 10^−3^ S/cm @ 55 °C) without SN introduction by increased segmental mobility of PEO in HSE [40,51]. In particular, the increase in ionic conductivity by SN introduction is prominent at room temperature.

### 3.4. Solid Electrolyte Interface (SEI)

**Figure 3:**
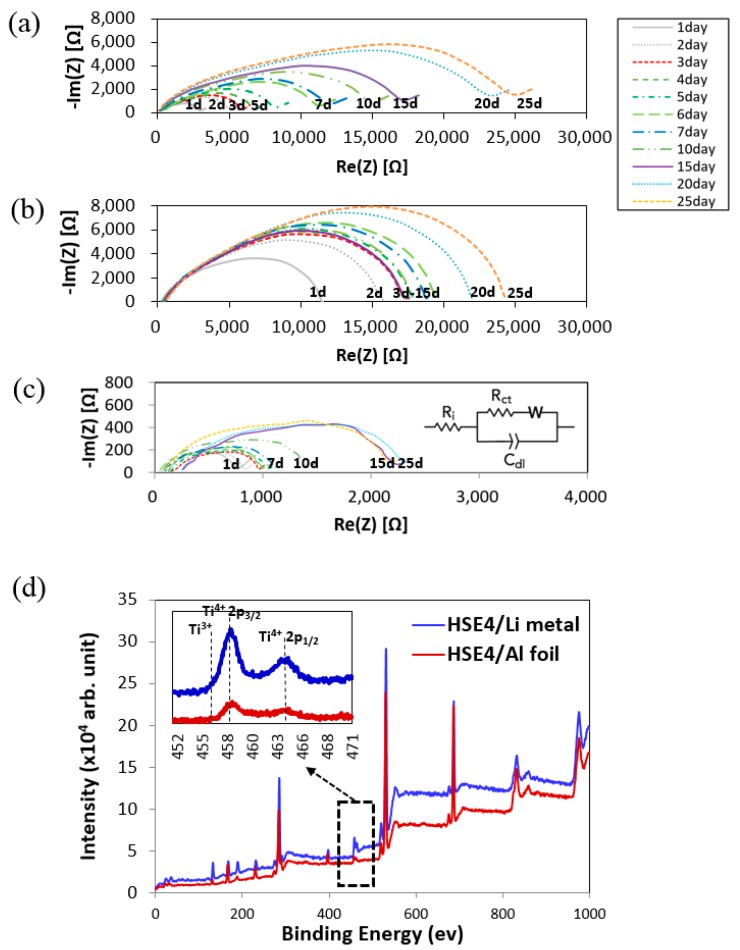
Impedance spectra of (**a**) Li/LATP/Li (**b**) Li/HSE-1/Li (**c**) Li/HSE-4/Li symmetric cells during 25 days (**d**) Ti2p_3/2_ and Ti2p_1/2_ photoelectron signals of Ti films (HSE-4) coated on Li metal and Al foil for 25 days.

Even with several controversies the LATP and Li metal is reported to react immediately upon a physical contact and forms an unfavorable solid electrolyte interface (SEI) layer on the Li metal surface [52,53]. Through this unfavorable phenomenon Li ion movement is reduced, thus decreasing the ionic conductivity. However, this can be suppressed by surface modification using PEO coating. The role of PEO in HSE is, at the same context, surrounding each LATP particle to protect the Li metal surface. The efficacy of the designed HSE on Li metal anode is investigated by measuring the AC impedance (Figure 3c). The chemical reactions of the designed model cells symmetrically composed of Li/LATP pellet/Li and Li/HSE/Li are evaluated for 25 days. The LATP pellet was made without sintering LATP powder after pressing. The experiment is performed to analyze the chemical reaction at the interface of LATP, HSE-1(PEO), HSE-4 and Li metal. Thus, the initial interfacial resistance of Li/LATP/Li symmetric cell was measured to be high. Among them, selected results of Li/LATP/Li, Li/HSE-1/Li and Li/HSE-4/Li are shown Figure 3a–c. The increase in the interfacial resistance has been observed in the system of LATP/Li metal [54] and PEO/Li metal by the characteristic chemical reactions [42].

The interfacial resistance of the symmetric cells designed as Li/LATP/Li and Li/HSE-1/Li continuously increase the interfacial resistance for 25 days. However, the resistance of the Li/HSE-4/Li cell is almost similar at a low value for all 25 days (Figure 3c). The result suggests that the reaction of HSE-4 on Li metal is far more effective than that of LATP. An equivalent circuit to describe the observed AC impedance spectra is shown in the inset of Figure 3c, which represents a hybrid solid electrolyte sandwiched between two blocking electrodes. In this equivalent circuit, *R*_ct_ is the bulk resistance of the solid electrolyte, *C*_dl_ (constant phase element) denotes the bulk capacitance of the hybrid solid electrolyte, and *R*i corresponds to the double layer capacitance at the electrode/electrolyte interface. Constant phase elements rather than capacitors were employed to describe non-idealities in AC impedance responses. The experimental data were fitted using the Randles equivalent circuit. Indeed, the AC impedance of a lithium metal symmetric cell is composed of the resistance at the electrolyte/electrode interface and the Ohmic resistance of the electrolyte itself.

In order to evaluate the impedance aforementioned result, X-ray photoelectron spectroscopy (XPS) measurements of the HSE samples are performed before and after the reaction with Li metals for 25 days (Figure 3d). For this, Ti peaks of HSE-4 pasted on Li metal after aging procedures for 25 days (blue line) are compared with HSE-4 on Al foil, with the physical substrate (red line) employed as a standard. Doublet peaks consist of Ti 2p_1/2_ and Ti 2p_3/2_: two strong Ti^4+^ peaks, i.e., 2p_1/2_ (Ti^4+^, 464.6 eV) and 2p_3/2_ oxide (Ti^4+^, 458.8 eV), two Ti^3+^ peaks (457 eV and 463.1 eV) [55].

Even with physical contact of HSE-4 with Li metal, the binding energy of two samples in this study is same. The resulting XPS has no difference where the Ti 2p_1/2_ and Ti 2p_3/2_ remain at the same energy position, indicating there is no Ti reduction (from Ti^4+^ to Ti^3+^) [54].

### 3.5. Electrochemical Stability

**Figure 4:**
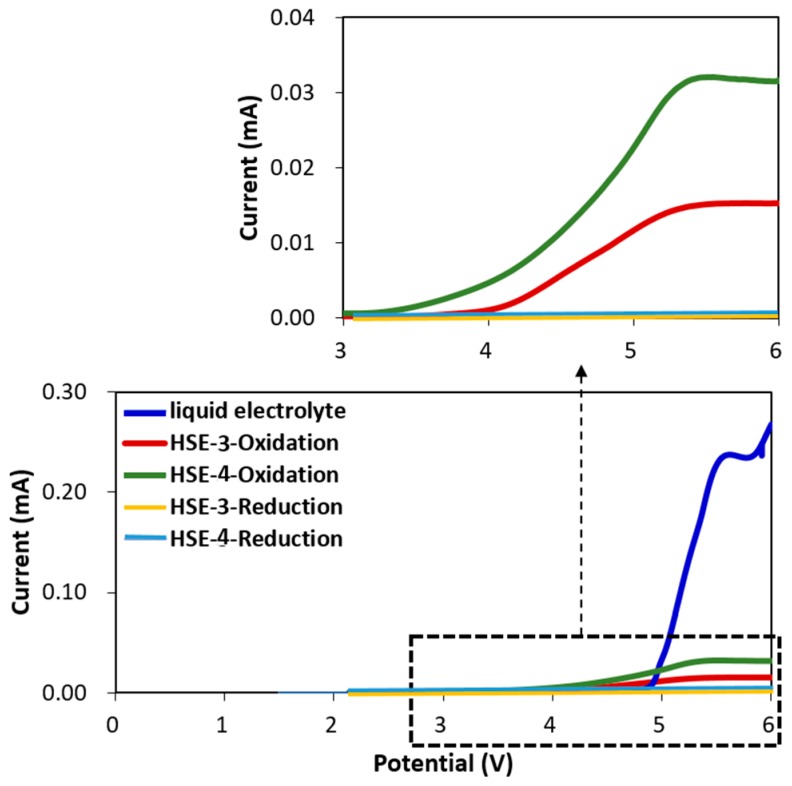
Linear sweep voltammetry (LSV) scans of HSE-3 and HSE-4. Pt is employed as a working electrode and Li metal is employed as reference electrodes. Scan rate is at the 1 mV/s.

To investigate the electrochemical stability of each electrolyte layer, linear sweep voltammetry (LSV) was measured from 1.5 to 6 V for both reduction and oxidation procedures (Figure 4). The oxidative stability is measured from the open current voltage (OCV) by sweeping up the voltage, while the reduction is measured by sweeping down the voltage. In Figure 4, the voltage was swept from ~2.2 V up to 6 V and then down again to 2.2 V. Both electrolytes HSE-3 and HSE-4 clearly show an onset of oxidation around 4 and 3.4 V, respectively. After going up to 6 V, the electrolyte will be partially or completely decomposed. During the sweeping down from that stage, the reductive stability was measured in a separate experiment: 1.2 mol L^−1^ LiPF_6_ in a 15:50:35 (in volume ratio) mixture of ethylene carbonate (EC) and ethyl methyl carbonate (EMC) and dimethyl carbonate (DMC) as the liquid electrolyte used as a standard contrast is oxidized at 4.8 V indicated by the sudden increase in the current (blue line). HSE (HSE-3) and HSE+SN (HSE-4) are also oxidized even if the absolute current intensity is relatively lower than that of liquid electrolyte. This electrochemical investigation of the designed cells indicates protective film formation on the cathode surface in the first cycle which works as a solid electrolyte interphase (SEI) in liquid electrolytes. On the other hand, during the reduction procedure of both HSE-3 and HSE-4, the current is very low without noticeable peak in all voltage regions observed in this study.

### 3.6. Property of the Designed Electrolytes

**Figure 5:**
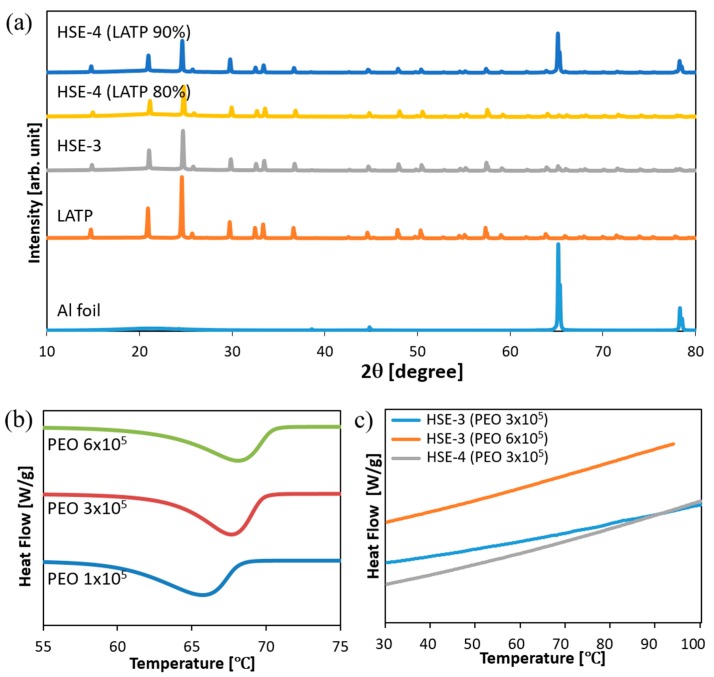
(**a**) XRD Patterns of LATP, HSE-3, HSE-4 (**b**) DSC of PEO with different molecular weight 1 × 10^5^, 3 × 10^5^ and 6 × 10^5^ (**c**) DSC of HSE-3 (PEO 3 × 10^5^), HSE-3 (PEO 6 × 10^5^) and HSE-4 (PEO 3 × 10^5^) composed of each PEO.

Due to inherent high boundary resistance, LATP hardly generates high ionic conductivity. A value of ca. 3.4 × 10^−3^ S/cm at 293 K, is among the highest conductivities reported for LATP-based systems [56,57,58]. To overcome this disadvantage, PEO is employed in this study. The structure of the PEO-incorporated system prepared on Al foil is investigated using X-ray powder diffraction (XRD) in Figure 5a. The XRD patterns of LATP-incorporated systems are almost similar. XRD patterns of HSE films exhibit lower intensity by the polymer incorporation without peak broadening. With the SN incorporation, XRD peak intensity becomes far lower, indicating the dilution of the whole system. Nonetheless the lattice structure is effectively maintained.

The ionic conductivity and thermal characteristics are modified according to the molecular weight of the PEO, which changes from 1 × 10^5^, 3 × 10^5^ to 6 × 10^5^ in this study. The segmental mobility of PEO is investigated using differential scanning calorimetry (DSC) (Figure 5b). With the increase of PEO molecular weight, the peak temperature increases from 65.741 °C (PEO *M*_w_ = 1 × 10^5^), 67.663 °C (PEO *M*_w_ = 3 × 10^5^) and 68.157 °C (PEO *M*_w_ = 6 × 10^5^). In addition, the magnitude of the heat density decreases from 178.31 J/g, 172.41 J/g to 170.87 J/g. However, the designed HSE systems with LATP and SN do not generate characteristic peaks at the given condition in Figure 5c. This indicates that incorporation of LATP and SN effectively broadens the amorphous state to the wide temperature ranges, increasing the ionic conductivity under that condition. The DSC result of the system consisting of LiTFSI has been verified to become amorphous when the ratio of the [EO]/[Li] is higher than ten [59]. In this study, the system consisted of LiTFSI with the ratio [EO]/[Li] = 8 exhibits an amorphous phase with the help of SN introduction. Based on this ionic conductivity result, the system with higher molecular weight PEO *M*_w_ = 6 × 10^5^ is selected to obtain the enhanced mechanical property for the design of HSE, while lower molecular weight PEO *M*_w_ = 3 × 10^5^ is employed for better mixing efficacy to design the cathode electrode.

### 3.7. Capacity and 1st Coulomb Efficiency of the Designed Coin Cells

**Figure 6:**
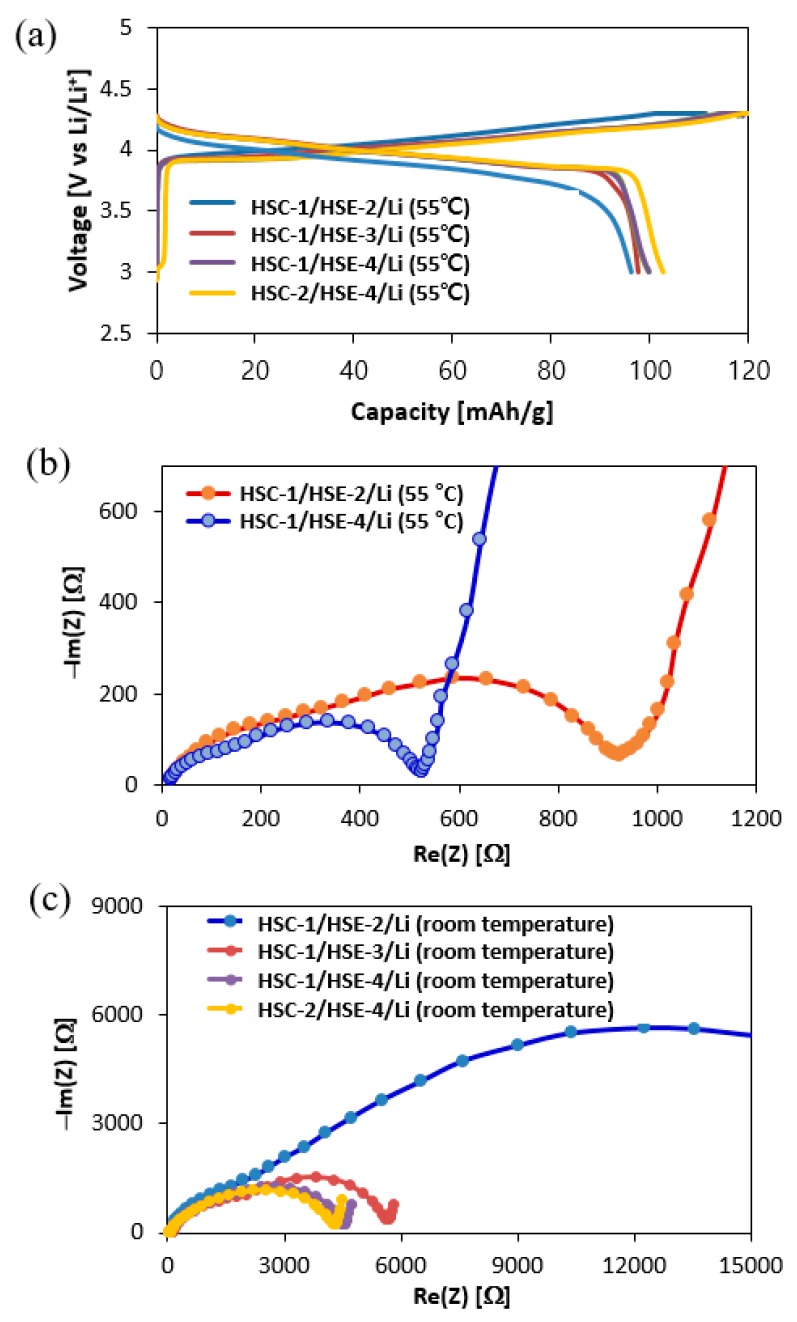
(**a**) 1st charge-discharge profiles of HSC-1/HSE-2/Li, HSC-1/HSE-3/Li, HSC-1/HSE-4/Li and HSC-2/HSE-4/Li at 55 °C. EIS profiles of (**b**) HSC-1/HSE-2/Li and HSC-1/HSE-4/Li at 55 °C and (**c**) HSC-1/HSE-2/Li, HSC-1/HSE-3/Li, HSC-1/HSE-4/Li and HSC-2/HSE-4/Li at room temperature.

Figure 6a shows specific capacity-voltage curves of the first charge-discharge cycles of the selected model cell systems. The 1st cyclic profiles of the LCO-LMO/HSE/Li system for each designed HSC-1/HSE-2/Li, HSC-1/HSE-3/Li and HSC-1/HSE-4/Li and HSC-2/HSE-4/Li are compared at 55 °C. The HSC-1/HSE-2/Li curve has more polarization loss compared to other curves. Further, the plateau region is quite broader for three other cases compared to the discharge curve of HSC-1/HSE-2/Li in terms of the slope nature. Although the difference is small in all the systems, the result of HSC-1/HSE-2/Li shows slight a disadvantage compared to the three other systems. Electrochemical Impedance Spectroscopy (EIS) results at 55 °C (Figure 6b) and room temperature (Figure 6c) are shown. LiPF_6_ is generally used for liquid electrolytes, while LiClO_4_ and LiTFSI are favorably employed for solid electrolyte systems. The ionic conductivity of the designed HSE is not so different (Table 1) but the resistance and charge-discharge efficacy in the designed Li ion cell are very different. LiPF_6_ (HSC-1/HSE-2/Li) and LiTFSI (HSC-1/HSE-3/Li, HSC-1/HSE-4/Li& HSC-2/HSE-4/Li) are used with PEO in this study, in which the physical properties of HSC-1/HSE-2/Li are different from other three systems.

However, for LiPF_6_ (HSC-1/HSE-2/Li for this study) and LiTFSI incorporated systems (HSC-1/HSE-4/Li for this study), the resistance of the system in actual Li ion cell state doubles at 55 °C and LiPF_6_ (HSC-1/HSE-2/Li in this study) and in LiTFSI incorporated systems (HSC-1/HSE-3/Li for this study); the resistance of the system in the actual Li ion cell state increases four-fold compared with that at room temperature. In the Li ion cell state, the re-crystallization kinetics of PEO has been effectively slowed by the use of Li ions and bulky anions [60]. The moisture generated by the electrode-cooling, decomposes LiPF_6_ into PF_5_, leading to the resistance increase by the interaction between PEO and SEI [61]. Through this effect, in addition to overcharging, the constant voltage region becomes broader during the charging process while capacity decreases during the discharging procedure. When moisture-resistant LiTFSI or LiTFSI+SN are used, aforementioned phenomena are reduced and capacity can be increased. In the system composed of LiTFSI+SN (HSE-4), SN promotes the Li ion dissolution, thus capacity increases far higher than that of LiTFSI-only (HSE-3). The charging-discharging process occurring at room temperature also shows similar results for the three systems while the HSC-1/HSE-2/Li composed of LiPF_6_ exhibits a dramatic difference. This result indicates that the moisture formation after the cooling procedure turns the LiPF_6_ into PF_6_ gas and SEI in PEO, where SEI decreases the efficiency of the battery function significantly.

The Li ion transport resistance in the HSE system is determined by its through-plane resistance instead of the ionic conductivity. The actual Li ion transport resistance may increase with increasing HSE thickness [62]. The designed HSEs in this study are the same thickness. Therefore, the higher conductivity of HSE+SN(HSE-4) ensures excellent Li ion transport performance. This is due to higher ionic conductivity of HSE-4 than HSE-3 and HSE-4 (Table 1), and the reduced resistance of a designed system even with the same thickness (Figure 6c).

**Figure 7:**
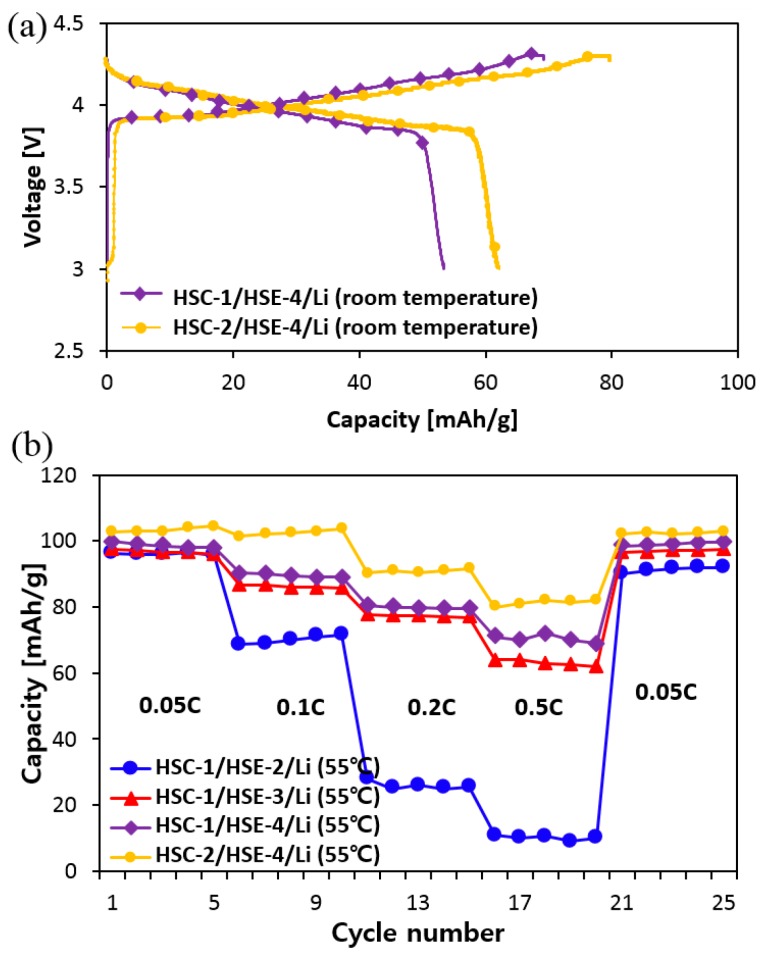
(**a**) 1st charge-discharge profiles of HSC-1/HSE-4/Li cell and HSC-2/HSE-4/Li cell at room temperature. (**b**) C-rate performance of HSC-1/HSE-2, HSE-3, HSE-4/Li cells and HSC-2/HSE-4/Li cell at 55 °C.

Figure 7a displays 1st charge-discharge profiles of the LCO-LMO/electrolyte/Li metal systems of selected HSC-1/HSE-4/Li and HSC-2/HSE-4/Li (both are based on LiTFSI+SN composition) at room temperature. This indicates that SN addition boosts the battery function compared with the cells employing LiPF_6_ (HSC-1/HSE-2/Li) and LiTFSI-only (HSC-1/HSE-3/Li), which are not working effectively at room temperature. The difference is clearer by the C-rate performance at 55 °C (Figure 7b). The C-rate values of each region are on the graph: 0.05 C, 0.1 C, 0.2 C, 0.5 C, and 0.05 C. At the 1st charging procedure, the capacity of each cell is 111.4 mAh/g (HSE-2), 118.5 mAh/g (HSE-3), 118.9 mAh/g (HSE-4), and 123.4 mAh/g (HSC-2), while at the discharging procedure, it is 96.2 mAh/g, 97.6 mAh/g, 99.9 mAh/g, and 102.7 mAh/g, respectively. At the initial formation, Coulombic efficiency was 86.4%, 82.3%, 84.0%, and 83.2%, respectively, based on the ratio of discharged capacity to charged capacity. In addition, their Coulombic efficiency increases continuously and is stabilized. After the initial cyclic repetition, the overall cyclic repetition over the 99% is maintained. After 20 cycles, it is also maintained by the charging-recharging at 0.05 C.

## 4. Discussion and Conclusions

In this study, hybrid solid electrolytes (HSEs) composed of LATP, PEO and SN are successfully designed without protection layer introduction. The solid electrolyte, LATP interaction with Li ion, promotes the reduction of Ti^4+^ into Ti^3+^. The LATP shows ion conductivity of 3 × 10^−3^ S/cm under the state of Ti^4+^ but it becomes far lower under the state of Ti^3+^ due to structural deformation affecting Li ion movement [11]. We observed no reduction of Ti^4+^ into Ti^3^ in the designed system which is investigated by Electrochemical Impedance Spectroscopy (EIS) and X-ray photoelectron spectroscopy (XPS). 

In addition to the new hybrid solid electrolyte (HSE), new hybrid solid cathodes (HSCs) are designed and investigated, composed of LATP, PEO, LiTFSi and SN. Typically employed for mass-production, slurry casting methods are utilized followed by lamination to reduce contact resistance. In addition, during the dynamic charge-discharge procedure of the designed Li ion cells, the effects of internal resistance on the charge-discharge procedure and C-rate changes are investigated. The interfacial resistance of HSE-4/Li metal symmetric cell is greatly improved compared with the LATP/Li metal symmetric cell (Figure 3). This suggests that the solution casting process and lamination introduced in this study lower the contact resistance between the Li metal and HSE-4. With good cycling stability, the designed cell also exhibits reasonable interfacial contact efficiency with electrode. 

We suggest the new electrolyte system is advantageously utilized in all solid-state Li batteries. Even without any protection layer, the designed system shows no reduction of Ti. This study contributes to a new design technology and further possible mass-production of all solid-state Li batteries in more economical and effective ways.

## Figures and Tables

**Table 1:** Designed hybrid solid electrolyte (HSE) and hybrid solid cathode (HSC) in this study.

		LATP (g)	PEO * (g)	LiPF_6_	LiTFSI	SN (g)	LMO+LCO (g)	EO:Li (Mole ratio)	Ion. Con. @23 °C [S/cm]	Ion. Con. @55 °C [S/cm]
HSE	HSE-1	-	100	●	-	-	-	8:1	1.7 × 10^−7^	-
	HSE-2	80	20	●	-	-	-	8:1	1.2 ×1 0^−4^	2.4 × 10^−3^
	HSE-3	80	20	-	●	-	-	8:1	1.5 × 10^−4^	1.4 × 10^−3^
	HSE-4	80	20	-	●	5	-	8:1	2.0 × 10^−4^	1.6 × 10^−3^
HSC	HSC-1	12.8	12.8	-	●	-	30 + 30	10:1	-	-
	HSC-2	12.8	12.8	-	●	5	30 + 30	10:1	-	-

The ionic conductivities of HSE at 23 and 55 °C are shown. * PEO *M*_W_ = 6 × 10^5^ g·mol^−1^, is used for HSE-1 to HSE-4, while PEO *M*_W_ = 3 × 10^5^ g·mol^−1^ are used for HSC-1 and HSC-2. For the whole battery design, HSE-1 to HSE-4 are combined with HSC-1, but HSC-2 is only combined with HSE-4.

## Supplementary Files

Supplementary File 1
